# One uncertainty added on top of another: Challenges and resources of mothers of preterm infants during the COVID-19 pandemic

**DOI:** 10.3389/fpsyg.2022.968192

**Published:** 2022-09-29

**Authors:** Palmor Haspel Shoshi, Rivka Tuval-Mashiach, Alona Bin Nun

**Affiliations:** ^1^Shaare Zedek Medical Center, Jerusalem, Israel; ^2^Herzog Academic College, Jerusalem, Israel; ^3^Department of Psychology, Faculty of Social Sciences, Bar-Ilan University, Ramat Gan, Israel

**Keywords:** stress, coping, challenges, resources, COVID-19, mothers, preterm (birth), neonatal intensive care unit

## Abstract

**Aims and objectives:**

To qualitatively explore COVID-19-related experiences of mothers of preterm infants in the Neonatal Intensive Care Unit (NICU), the main challenges they face, and the resources available for them.

**Background:**

The birth of a preterm infant is a stressful event under otherwise normal circumstances. The outbreak of COVID-19, the uncertainty about the virus and how it spreads, and the restrictions imposed, may have exacerbated the stress of caring for a preterm infant.

**Design:**

Retrospective interviews.

**Methods:**

In-depth interviews with 12 mothers of preterm infants who were hospitalized in the NICU at the time of study. The interview addressed challenges and resources related to coping with the pandemic. The interviews were transcribed and content analyzed, based on Lieblich et al’s model for narrative analysis. This research was conducted in accordance with the COREQ checklist.

**Results:**

The overarching experience shared by all mothers was accumulative stress caused by a combination of factors related to the infant’s health and COVID-19-related stressors. A central theme was the dissonance between the mothers’ expectations from the birth and infant, and the reality they encountered. Other themes included fear of infecting the infant, loneliness, and stress caused by the restrictions that disrupted daily routines. Resources included a sense of shared fate regarding the pandemic, improvements in the infant’s condition, religious faith, emotional support from the partner, and support from professional staff.

**Conclusion:**

Caring for a preterm infant during a pandemic is a challenging experience on many levels. The loss of significant support resources puts mothers of these infants at a higher risk for psychological distress.

**Relevance to clinical practice:**

Awareness of mothers’ accumulative stress due to the COVID-19 pandemic may assist the staff in developing procedures that can alleviate parental stress, for example by enabling mothers to connect to each other, giving clear information to compensate for physical and social distancing and providing professional mental health support.

## Introduction

All parents dream of a healthy infant. During pregnancy, parents have expectations and hopes for a healthy, perfectly formed infant. When a preterm infant is born, parents experience acute stress due to the unexpected event and the change in their parenting role (Al Maghaireh et al., 2016), and anxiety about the precariousness of their infant’s situation (Hagen et al., 2019). They are fearful for the infant’s wellbeing (Bry and Wigert, 2019), pained by seeing their infant suffer, struggle to forge a connection with their infant and possibly even with his or her appearance (Agrawal and Gaur, 2016; Spinelli et al., 2016), while at the same time mourning the premature end of the pregnancy (Obeidat et al., 2009; Valizadeh et al., 2013).

Since the outbreak of COVID-19 in 2019, the prevalence of psychiatric disorders and loneliness among women was higher (Li and Wang, 2020). In the Neonatal Intensive Care Unit (NICU), parents of preterm infants have had to cope with additional stressors related to the pandemic, whose end is not in sight (Kluge, 2020), and with a tangible and life-threatening virus that requires social distancing (The Center for Disease Control and Prevention, 2022). Dubey et al. (2020), mentioned fear and anxiety concerning a new and little-understood disease, fear about one’s own health and that of one’s loved ones, worry about isolation should one become ill, and loss of supportive resources. Nurturing experiences such as breastfeeding, kangaroo care, and talking with the newborn infant, which routinely mitigate stress, may occur less frequently under these circumstances (Erdei and Liu, 2020). In addition, the parents had less opportunities to learn about and develop their self-confidence to care for their infants. The preparation for the discharge was carried out in the setting of basic care, when the discharge was imminent (Osorio Galeano and Salazar Maya, 2021).

Hobfoll (1989) presented the Conservation of Resources (COR) theory as a framework for understanding how people use resources differently to cope with stress. According to this theory, people aim to preserve, protect, and construct resources that help them cope. The stress of giving birth to a premature infant can be described as a sudden and unexpected loss of highly valuable resources. The loss of resources can cause psychological distress in the mothers of these infants, which can affect the infants’ medical outcomes (Shani-Sherman et al., 2019). Research on the psycho-social factors that affect parents with NICU infants is associated with severe parental distress during hospitalization and with a worse outcome for the infant’s future cognitive and emotional development (Grunberg et al., 2019). There is a wealth of research literature about the stressors involved in parenting a preterm infant. Thus, it is important to understand NICU parents’ psycho-social needs, particularly during the COVID-19 pandemic, not only to alleviate their distress during their hospital stay, but also to improve the long-term outcome for their infants (Hall et al., 2017; Bry and Wigert, 2019). In a recently published study, we (Bin-Nun et al., 2021) found that COVID-19 intensified the difficulties experienced by mothers of preterm infants and harmed their familial and social relationships. Most mothers reported feeling stressed, lonely, and helpless. In the absence of efficient coping strategies, anxiety levels and depression may rise. Moreover, maternal distress may be intensified if families must be separated and quarantined (Brooks et al., 2020; Osorio Galeano and Salazar Maya, 2021).

An infant’s medical condition may require neonatal isolation that is not related to COVID-19. During this time, the medical staff and the parents take extra precautions (e.g., wearing gloves and gowns at all times). COVID-19 created a new reality in which either the neonate or the parents may be exposed to the virus, necessitating further maternal isolation from the infant, whose condition may be life-threatening. This was one of the most extreme stressors encountered in the NICU as a result of the pandemic.

In light of the literature reviewed above, on heightened maternal stress due to COVID-19, the purpose of the current study is to investigate the challenges experienced by mothers who stayed in the NICU with their infant during the first weeks of the pandemic. We were interested in learning about the main challenges they encountered, how they perceived the medical staff’s performance, and the resources they used to cope with the circumstances created by COVID-19.

## Materials and methods

### The study approach

Due to our aim to focus on mothers’ lived experience and to learn on the challenges created by the pandemic, we adopted a qualitative/narrative approach. Because we wished to gain understanding of a phenomena that was new at the time, and due to the relatively low number of potential participants, we chose a qualitative approach. The narrative approach was deemed appropriate as its focus is on viewing narration as a meaning making process (Lieblich et al., 1998). This approach claims that people construct their life stories based on their lived experiences through a dynamic process, and that these stories shape and are shaped by their identities, as well as by cultural and social circumstances (Clandinin, 2006).

### Setting and sample

The participants in this study were 12 mothers of preterm infants, who delivered and were hospitalized in a large, Level III NICU during March–April 2020. The NICU admits approximately 1,000 sick neonates per year, and serves a general population of nearly 18,000 deliveries per year. There are a limited number of one mother/infant dyad in full rooming in, while most mothers are housed in 2–3 patients’ rooms. Since the COVID-19 pandemic started, many hospital policies were modified or adjusted. Babies were allowed to breastfeed at the breast and were not separated from a mother with COVID-19. Mothers were instructed to wear mask at all time during the hospital admission except for brief periods of eating or washing, and were instructed to wash their hands prior to any kind of baby handling. Distancing of at least 2 m between babies was implemented at all times. No more than one visitor at a time was allowed, and only belonging to the same nuclear family (spouse or child). Grandparents were not allowed to come into the NICU. If one parent was sick and the other in isolation, then the preterm infant remained alone without the presence of a family member. Based on Robinson’s (2014) considerations on the sample universe and homogeneity, the strategy chosen for data collection was purposive sampling technique (PS, Etikan et al., 2016). The rationale for the PS technique was to explore the experience from diverse perspectives, that we saw as relevant to the researched experience, and referred to the level and type of isolation of the baby in the NICU. Therefore, we sampled mothers who represented three different groups: mothers whose infants were isolated for medical reasons other than COVID-19, mothers who were separated from their infants because of suspected exposure to COVID-19, and mothers who were not separated from their infants at all. The mothers’ average age was 27 (*SD* = 6). All were married, 11 were Jewish, one was Muslim, and the majority of them (*n* = 9) were religious (orthodox or ultra-orthodox). The mothers had given birth to 0–9 children before the study (*M* = 1; *SD* = 3). Seven mothers were primipara. The length of hospitalization in the NICU was 7–176 days (*M* = 60; *SD* = 45).

Table 1 shows the demographics of the mothers and their infants.

**TABLE 1:** Details of the mothers and their infants’ condition.

Group	Participant	Maternal age	Sex of newborn	Delivery number	Pregnancy week	Single/twin	Birth weight	Hospitalization (days)
No isolation (NI)	9	25	M	3	27.4	S	1.095	89
	10	33	M	1	34.3	S	2.455	21
	11	30	M	1	29.0	S	1.200	43
	12	25	M	1	32.0	S	2.110	48
Medical isolation (MI)	1	41	F	10	27.0	S	1.150	71
	2	21	F	1	35.4	T	2.018	18
	3	22	F	1	30.4	T	1.440	40
	4	24	M	1	25.0	S	840	176
COVID-19 isolation (CI)	5	22	F	1	35.6	T	2.740	7
	6	21	F	2	28	S	3.065	92
	7	29	M	2	27.6	S	945	73
	8	30	F	2	34.1	S	1.950	46

#### Interviews and procedure

Mothers of preterm infants in the NICU were invited to participate in the study, in order to learn about their experiences in the NICU. Upon agreeing, they signed an informed consent, and were invited to a quiet room in the NICU. Participants were asked about their experiences since their infant were born and admitted to the NICU. Specifically, they were asked to describe their thoughts, feelings, and what they perceived as the challenges created by their infant’s condition and how COVID-19 impacted their experience. We asked about their strategies for coping with the situation, and what they found most effective. A two-stage narrative interview was conducted with all participants. The two-stage narrative interview was open-ended, and based on the narrative approach. Each interview lasted 30–45 min. All the interviews were held face-to-face, conducted in Hebrew, recorded and transcribed. Participation was voluntary and participants did not receive compensation for participation.

#### Analysis

The interviews were content-analyzed by two of the authors separately, based on the model for narrative analysis proposed by Lieblich et al. (1998). Each searched for stressors and resources that emerged in the narratives. Following the initial analysis, the authors met to discuss the emerging themes, and reached an agreement on the main themes identified. For the purpose of writing the manuscript, verbatim sections were translated from Hebrew to English for citations, translation was conducted by a bilingual English editor, fluent in both Hebrew and English.

#### Ethical considerations

The study was approved by the local IRB. Mothers of infants admitted to the NICU signed an informed consent form after receiving an explanation about the study. Participants were assured of their ability to leave the study at any time, or refuse to respond to any question in the interview. We paid careful attention to the sensitive topic of the interview and to the stressful conditions of mothers at the time of interview. First, mothers who agreed to participate were free to choose the time of interview as the interviewer was available in the NICU. Second, the interviewer is a trained psychologist with much experience in working with mothers of preterm babies, and in case of distress could assist in turning participants to get support. The interviewer was not the direct therapist of participants. Importantly, as two of the authors (S.P and A.B.N) are part of the NICU staff and therefore had met the participants at the NICU before beginning the study, the third author served as an external reader of the interviews, as she had no acquaintance with the participants.

## Results

The overarching theme that emerged from the interviews was the continuous need to adapt to an ever-changing reality when faced with multiple uncertainties related both to the infant’s condition and the COVID-19 situation. There was no difference in experience and distress described by mothers between those whose infants were at more risk than others, or whose infants required medical or COVID-19-related isolation. Most narratives described typical challenges that began from the moment of delivery. The mothers described a sense of everything being new and unexpected, even when the preterm infant was not their first child (as was the case for about half the participants). Three themes emerged from the interviews—the gap between expectations and reality; new and unforeseen challenges created by COVID-19-related restrictions; and resources that helped mothers cope with the COVID-19 situation.

### Expectations vs. reality and the need to constantly adapt

All mothers described a significant dissonance between their expectations and the reality forced upon them. They expected a normal delivery, but complications resulted in preterm delivery and infants who were admitted to the NICU. Mothers described how they had imagined their pregnancy and their infant, and how they had to adapt instantly to a very different reality. They felt that their role as mothers became secondary to the role of the medical staff, who took over responsibility for managing the infant’s care. In some cases, there were cautionary signs during pregnancy that enabled the mothers to anticipate certain complications. In other cases, the newborn infant’s need for special care and treatment came as a total surprise. In the latter case, the lack of opportunity to mentally prepare for the challenges posed by having an at-risk infant created even more stress for the mothers, and required their swift adaptation to a dangerous and intense reality, while still recovering from delivery. Elza, who experienced bleeding during the early months of her pregnancy, was told that she would likely give birth a little early, but the delivery at 27 weeks came as a complete surprise:

It was a shock. I told my husband to go and be with our older child, so he left, and then, within a few minutes, I gave birth. It was fast and surprising, everything happened so fast, the baby was taken to the NICU… Wait a minute, I need to process that I gave birth. But immediately the doctor from the NICU starts talking to me while I’m still in the delivery room. It took us a few days until we managed to take the element of shock out of our situation.

The narratives make it clear that the mothers had specific, often unconscious, notions about what should have happened during labor, when bringing home a healthy infant, during their maternity leave, etc., and all these expectations were abruptly shattered, not only because of the infant’s condition, but as we illustrate below, because of the COVID-19 pandemic as well. For example, mothers imagined celebrating the delivery with others during the first days after the infant’s birth. Mariam, a first-time mother said:

“Before [I gave birth], I imagined that I’d give birth, have many visitors, and a room full of flowers. Then, suddenly, no one was allowed to come. It sucks.”

Another mother, Martha, said:

“We were imagining all our friends coming to our home, and we planned a baby party, but now, all we can do is send pictures because they can’t even come and see him… the most difficult thing is to cope with the expectations and dreams that didn’t come true, coping with ‘how it should have been’…coping with the reality that everything is totally different, and managing to survive it and stay positive.”

Efforts to quickly adapt to the new reality were hampered by constant uncertainty, caused by the infant’s unstable and dynamic condition, as well as by the rapidly changing regulations related to the COVID-19 pandemic. This study was conducted during the first months of the pandemic, when the restrictions were unclear and changed from 1 day to the next. For example, partners, who were initially allowed to stay in the NICU with the mothers or to replace them, were suddenly not allowed in the NICU in order to minimize the risk of contagion. In another case, one of the infants in the NICU was suspected of having contracted COVID-19 and all parents of the infants who shared the same room were sent home and not allowed to see their infants for a few days. One of the mothers said that any time the ground started feeling firm beneath her feet and she started to adapt, everything was shaken up again and she had to readjust to new COVID-19 restrictions. Another mother described her experience as “one uncertainty on top of another.”

### Challenges related to COVID-19

All the participants described in detail the many challenges and difficulties they encountered because of COVID-19. We distinguished between practical and emotional challenges for purposes of clarity, although the participants described them together and as interrelated.

#### Practical challenges

The COVID-19 pandemic created practical challenges that added to the burden of coping with a premature infant. All the mothers described how the COVID-19 restrictions had made life more complicated and required more planning, adjustment, and constant improvisation. One mother, Elana, described how she could not visit her baby whenever she wanted to, because public transportation suddenly stopped without any prior notice due to the high rates of contagion in public places. Another mother, Olivia, said that they had been due to be released from the hospital to continue the preterm infants infant’s treatment in the community, but because community medical services were closed due to the lockdown, the doctors decided to keep them in the NICU for another few weeks so that the preterm infants preterm infants could get the treatment he needed. Another practical challenge was the need for parents to take extra precautions and wear special gowns in the NICU, which was not easy and was experienced as interfering with the parent’s ability to bond with the baby. Nicole said:

All these ungainly layers, this gown and that gown, and the masks. It is annoying, and you have to keep social distance and wear a mask, and I feel that my baby doesn’t see me smiling at him because I’m always behind a mask.

A few mothers pointed out that the medical staff was more stressed and overloaded, especially when some staff members had to quarantine or take care of their own family members. They said that this affected the staff’s availability and patience toward the parents. Several mothers said that they had planned on their own mothers helping them with their other children at home, while they stayed with their infant in the NICU, but the circumstances and the lockdown prevented them from receiving this help, and they did not know how to divide their time between home and the hospital. The stress of caring for the family at home and the infant in the NICU was mentioned by all participants who had other children. While most parents of infants who require special attention are torn between home and the hospital, the pandemic added difficulties with commuting, finding babysitters (which was prohibited during the lockdown), getting help from extended family, and running simple errands for the baby. When asked what was most difficult for her, Mira said:

That my parents can’t come to visit, and that we can’t be together. It’s very difficult for them too, not to see their grandchild, and when my husband was in quarantine I was here all alone, all the time. It was very difficult.

#### Emotional challenges

The tone common to all the narratives reflected emotional drain and exhaustion. The word “difficult” was the word most frequently used in all narratives. Mothers expressed concerns about their infant’s condition and development, and fears that they, their partners, or any of the healthcare providers would be a source of infection for their infants with COVID-19 and endanger them. This created a heavy sense of responsibility and anxiety. Charlene says:

I was terrified that I’d have to be in quarantine because of contact with a sick person, and I knew this meant my baby would be alone. I saw that all the others didn’t follow the instructions as carefully as I did, so I started to touch the doors with gloves, I didn’t touch anything. We stopped sitting in the family room near the NICU because I saw that other people touched the surfaces. There was a difference between me and my husband. I was hysterical, he was not, so I had to ask him to put gloves on. It was so tough, I really hit rock bottom. nothing could reassure me.

As expected, the infant’s condition had the greatest effect on the mothers’ moods and emotions. Premature infants are often unstable, and it takes time before their medical condition stabilizes. At the time of the interviews, about half the mothers still reported fluctuations in their infant’s health, and the others described the infant’s condition as more stable and progressing well. Nevertheless, all the mothers were still concerned about their infant’s medical condition, and described feeling emotional distress. These feelings were exacerbated by the pandemic for several reasons, including the inability to share their concerns face-to-face with other family members such as the father and the grandparents. This increased the mothers’ sense of bearing the burden of responsibility alone. This especially applied to partners, who could not stay in the NICU with their wives due to COVID-19 restrictions, or because they had to care for the other children at home. Many times, the partners were updated only after the changes happened or decisions were made regarding the infant. In addition, most mothers described a sense of loneliness resulting from the COVID-19 restrictions, due to the inability to have visitors, receive emotional and physical support from their own mothers or partners, or talk with other mothers in the NICU to support each other. Charlene, who gave birth right before the pandemic, was able to compare the time when her parents could come and be with them, with the time when they were asked to stay away:

When we were told that my parents couldn’t come anymore, I think that was the most difficult moment for me, because then, we were left alone. At the beginning we were very enveloped; my parents came to the NICU every day. It gave us time to breathe; for each of us to get some space… And then, suddenly, my parents weren’t allowed in anymore.

### Coping resources

As described above, the simultaneous, multiple stressors were overwhelming and ongoing. The mothers in our study had to find ways to cope with the circumstances, especially as it became clear that it was going to take a long time before their situation would change, both because of their infant’s condition and because of the spread of the pandemic. We asked the mothers what helped them cope with the stress and the uncertainty. They all reported that it took time and effort to find the right resources, mainly because the resources they used in previous stressful situations were no longer available. Two examples of resources that are often used to relieve stress were social support and having personal time to rest, be distracted from the situation, or “recharge their batteries.” These two resources were seriously compromised, as we described above, and therefore, mothers had to find other ways of strengthening themselves. Five resources that many of the mothers described were: improvement in the infant’s condition; religious faith; the ability to emotionally lean on their partners; feeling connected to the world and to other mothers with regard to the virus; and the support of the professional team at the hospital. The infant’s condition was described as the main contributor to facilitating or inhibiting their ability to cope with their situation. Improvements, even when small or temporary, were uplifting for the mothers and gave them hope and strength. Charlene says: “The only thing that could encourage me was my infant’s progress. Every time there was progress, I felt that there was a light at the end of the tunnel, and it encouraged us.”

Most of the mothers were religious, and therefore their religious beliefs served as a protective factor. Rose said:

I believe in God and it helps. I know that everything that happens should have happened, so if it happened to us, I’m telling myself it should have happened. I won’t say it’s not difficult. There’s tension and anxiety. But there’s something to hold on to. There is someone to turn to, I can pray to Him. That keeps me going during these times.

Despite the absence of the natural support system (e.g., friends, grandparents, sisters) during the pandemic, mothers still found ways to benefit from the support of their significant others. The majority of participants referred to their partners as their main source of support and as a central resource that enabled them to cope. Martha says: “This is something you can cope with only together.” She refers to the relationship with her partner as both affecting and being positively affected by their experience in the neonatal unit: “There is no doubt it strengthens our relationship.”

Significant and mutual relationships with other mothers in the unit were also mentioned as a meaningful resource, as was a sense of being connected to the world with regard to the pandemic. Many mothers described the notion of “being in a bubble,” in which outsiders cannot understand the drama and intensity of the life-threatening experience. The ability to share their experiences with others and get tips and encouragement from mothers who had already gained experience in the NICU was a major resource. In addition, the pandemic gave them a sense of sharing a common fate with the rest of the world. Donna says:

What helps me here is the company of other mothers and knowing that everyone is dealing with this pandemic together. Several mothers became real friends for me, and I feel much more comfortable talking to them about what happens with us than talking to my other friends, from the time before all this happened. Here, with everything that happens to my baby, those who didn’t go through the same thing will never understand. Only the mothers here can understand, so I can share with them, and I can let myself be weak with them, and they can be weak with me, and let go of everything that bothers them. And this is something that really helps me very much.

Finally, the professional staff was described in many of the narratives as contributing significantly to the mothers’ wellbeing and ability to cope. Relevant aspects of the staff’s behavior were the doctors’ patience and their availability to explain and attend to the parent’s need to know what was going on with their infant; the nurses’ ability to encourage the parents and instill hope in them; and the warm atmosphere in the unit. Elana said:

The medical staff and the nurses are amazing. There are wonderful nurses, you feel their embrace. You are in a very complex situation, and they try to encourage, to explain, always give hope. It feels good. We felt all along that he (the infant) was in good hands. Even when I had to go home and leave him behind—that was the hardest thing for me… I was relaxed because he was in good hands.

Another mother, Leona, described her difficult experience when she, as a new mother, did not take even short breaks for herself, because she was too stressed about needing to be with the infant all the time. She recalls how one of the nurses told her she had to go out and take a break, and assured her that it was the right thing to do. She says: “I couldn’t stop crying that day, I felt like I couldn’t take a break, it’s forbidden, and she (the nurse) said: ‘go freshen up, don’t worry.”

However, when one or more of the resources described above were missing or compromised, mothers described more distress and helplessness. Such was the case with Charlene, who referred to her inability to get the support she needed. She said: “I was in a difficult mental state, my husband tried to help me as much as he could, but at some point, he just couldn’t do it anymore. It was difficult.” Another mother said that when a nurse was impatient, it could ruin her day, and make her anxious and hesitant to ask questions.

The resources we described served to offset the many challenges and anxieties that are part of the complicated and multi-challenging experience of caring for a preterm infant during the COVID-19 pandemic. Although this study did not aim to quantify the mothers’ distress, the mothers’ narratives showed that those who were fortunate enough to have more resources available to them, were able to cope better and reported a more positive wellbeing.

## Discussion

Parenting preterm infants, and mothering in particular, has been found to be a major source of psychological distress (Heerman et al., 2005; Swanson et al., 2012; Rogers et al., 2013). Factors associated with maternal stress in the context of NICU hospitalization include the baby’s appearance; difficulty creating a bond with the infant (Bry and Wigert, 2019); developing maternal self-efficacy (Swanson et al., 2012); and being perceived as an outsider in the medical environment (Fenwick et al., 2001; Al Maghaireh et al., 2016). The outbreak of COVID-19 was assumed to significantly increase the emotional burden carried by parents of preterm infants (Osorio Galeano and Salazar Maya, 2021). Therefore, in the current study we aimed to explore the unique challenges faced by mothers of preterm infants, who were hospitalized in the NICU during the COVID-19 pandemic for an extended period of time, and the resources available for these mothers.

Our findings show that in addition to the well-documented challenges that usually characterize NICU hospitalization, the pandemic significantly increased the burden and the stress experienced, both by exacerbating existing challenges inherent to the hospitalization, and by adding new challenges and depriving mothers of resources that would have been available to them under normal circumstances, such as their family’s social support. It has been documented that the pandemic had a negative psychological impact in general, even in populations which were not coping with emergencies (e.g., Li and Wang, 2020; Tull et al., 2020). Specifically relevant is Li & Wang’s large scale UK study, in which loneliness was reported in more than one-third of the participants. We suggest that stress and loneliness were exacerbated compared to the regular population, due to the additional burden of their baby’s situations, but also because the satisfaction of their needs was compromised due to the circumstances created by the lockdown and social distancing policies. Hobfoll’s theory of Conservation of Resources (COR theory) (2002) states that when faced with stressful situations, people seek to obtain, retain, and protect their personal and social resources. The loss or gain of a resource is the primary mechanism that drives stress reactions (Hobfoll, 1989). Based on the COR theory, the demands related to parenting an infant in the NICU were exacerbated by changes and restrictions related to the COVID-19 pandemic, which threatened personal and social resources and increased parental stress. Indeed, our findings point to the experience of extreme stress in the mothers. It seems that managing stress was especially difficult because of the threat of the pandemic, which was at an extremely high level during the first months before vaccinations were available, adding to their sense of helplessness regarding the infant’s life-threatening condition. Gaps between available stressors and resources were described regarding three aspects: lack of (sufficient) social support, both from professional team, from peer mothers, and from partners and the families. Mothers took extra cautionary steps that went above and beyond the hospital policies regarding social distancing. They stayed away from the other parents, and did not let other family members replace them in the NICU, even when this was possible. This strategy, which may have been necessary during the COVID-19 pandemic, resulted in the loss of social support by fellow mothers or by family members. Social support is considered one of the most significant resources for mitigating stress (Hobfoll, 2002). The social distancing imposed upon the mothers by the official restrictions and at their own initiative deprived them of natural support resources, resulting in a sense of loneliness, anxiety, and emotional burden. A recent study found that COVID-19 added new fears for parents of preterm infants, who perceived the virus to be a new threat for their vulnerable infants, and these parents had less family and professional support than under regular circumstances (Galeano and Maya, 2021). Additionally, mothers felt that they could endanger their infants by spreading the virus to them, as little was known at the time about mechanisms of contagion. On top of their distress, they were also under great stress and did not have the option of sharing experiences with peer mothers and other family members due to social distancing. The mothers had less opportunities to learn and develop confidence to care for their infant, the parenting’s care of the infant is determinant for his health and wellbeing, especially when the discharge was imminent (Osorio Galeano and Salazar Maya, 2021).

Finally, the ability to adapt to the situation was inhibited by the many unpredictable factors involved. One of the crucial factors for successfully coping with stressful situations is the ability to plan ahead to effectively manage available resources. Stressful, ongoing, and dynamic situations are hard to cope with (Ramezani et al., 2014). The mothers in our study had to cope with the daily policy changes and restrictions that were issued by the authorities and the hospital. This uncertainty, where restrictions escalated every day, made adaptation even more difficult, as it required constant adjustments at a time when mothers were already emotionally and physically vulnerable due to their infant’s unstable condition.

The long hospitalization in the NICU combined with the intense stress associated with the unpredictability, uncertainty, and uncontrollability of the situation and the loss of important social resources, may make mothers of preterm infants more prone to psychological distress, depression, and post-traumatic stress (Gangi et al., 2013). Recently, Erdei and Liu (2020) suggested that the combination of NICU-related parental stress and COVID-19-related challenges and constraints can have a stress contagion effect. In other words, parental stress may affect how parents engage and connect with their preterm infant, which can have a long-term effect on the infant’s development and on the family’s adjustment in general.

The participants in this study described several resources that they found helpful for coping with these stressors, including emotional support from their partners and from other mothers, a sense of shared fate regarding the pandemic, and support from professional staff. Another important factor was improvement in the infant’s condition, Finally, religious faith was a significant resource in our sample, which included mostly religious women. Religious faith was found in previous studies as well to be a protective factor (Yazarloo et al., 2020). The parents’ spiritual lens is relevant in an unfamiliar and unexpected hospital admission for their newborn, which is intensified during a pandemic (Brelsford et al., 2016).

### Practical implications

As the COVID-19 virus remains a major threat, it is important to develop strategies and services that can help parents, and especially mothers, manage stress during the critical period of NICU hospitalization.

Our findings suggest that there are ways in which the professional staff can assist mothers in alleviating their stress. First, it is important to maintain social support as much as possible by enabling mothers to connect to each other, even when social distancing restrictions are imposed. Second, staff support is a significant resource, and therefore should be considered an integral part of treatment. Despite the challenging reality, encouraging mothers’ early bonding with their infants, breastfeeding support, use of skin-to-skin care can lower mothers’ stress level (Melnyk et al., 2006). Furthermore, connecting new mothers with mothers with experience with NICU hospitalization, and referring mothers for supportive services in the community before discharge is important. Awareness of parents’ accumulative stress due to the COVID-19 pandemic may help the staff develop procedures for alleviating the shared trauma and stress caused by COVID-19 (Baum, 2014), for example, by enabling mothers to connect to each other, or providing professional mental health support. Third, a mental health professional should be available for parents, as well as staff members, who need additional support (Hall et al., 2017). Finally, since transparency offsets many of the challenges related to the instability and unpredictability inherent to the situation, it is highly recommended that the guidelines be made clear, and that parents be updated on all changes and restrictions related to the pandemic (Galeano and Maya, 2021). The COVID-19 pandemic created unique stressors, which may recur in the future, and therefore understanding the best ways to enhance and conserve resources for mitigating stress is crucial.

The study has several limitations. The sample is small and quite homogeneous, as most of the participants were religious. While this represents the population served by the hospital, it is hard to generalize our findings for non-religious populations, especially as religious belief was found to be a major resource. Since most of the mothers in our NICU led a religious lifestyle that is characterized by deep faith and a close-knit community (Dollahite and Marks, 2009), the study findings about available resources are especially relevant for homogenous communities with significant religious resources. Although the religious narrative eased their coping with the double stressors, yet during COVID-19 assistance from the community may be hindered.

It is important to interview mothers from different ethnic and religious backgrounds to expose other resources that are used. Furthermore, only mothers were interviewed in this study, as mothers and their partners have been shown to experience NICU hospitalization differently (Fegran et al., 2008). Interviews with fathers may yield different themes that could provide new insights on parental experiences in the NICU during the COVID-19 pandemic.

The study was conducted during the initial outbreak of the pandemic, and the parents meticulously adhered to rules regarding social distancing and preventing the spread of the pandemic. It is possible that over time, people would become habituated to living with COVID-19 and the anxiety level would be lower.

Despite this limitation and due to the persistence of the pandemic, it is important to note that the characteristics of the NICU examined in the current study are similar to those of other NICUs elsewhere, and therefore the stressors could be relevant for mothers or parents who are caring for a preterm infant in the NICU even under different circumstances or other environmental contexts, given that the pandemic is ongoing or worsening, or in other circumstances that create ongoing social and/or physical distancing. Therefore, we believe that the challenges we described in this qualitative in-depth exploration are shared by many mothers of preterm infants who have to cope with the COVID-19 virus and its impacts.

## Conclusion

Caring for a preterm infant during a pandemic is a challenging experience on many levels. The loss of significant support resources puts mothers of these infants at a higher risk for psychological distress.

### Relevance to clinical practice

Awareness of parents’ accumulative stress due to the COVID-19 pandemic may assist the staff in developing procedures that can alleviate parental stress, for example by enabling mothers to connect to each other and providing professional mental health support.

### What does this paper contribute to the wider global clinical community?

• This study raises awareness about the significant challenges of caring for a preterm infant during a pandemic, which may make mothers of preterm infants more prone to psychological distress.
• It is important to be aware of the coping resources that many of the mothers described, namely improvement in the infant’s condition; religious faith; the ability to emotionally lean on their partners, other mothers and the professional team at the hospital, and feeling connected to the world, to help improve the mothers’ wellbeing.
• The staff can contribute to developing procedures that can alleviate parental stress, for example enabling mothers to connect to each other and providing professional mental health support.

## Data availability statement

The original contributions presented in this study are included in the article/supplementary material, further inquiries can be directed to the corresponding author/s.

## Ethics statement

The studies involving human participants were reviewed and approved by the Shaare Tzedek IRB Committee. The patients/participants provided their written informed consent to participate in this study.

## Author contributions

PHS was responsible for data collection, data analysis, and writing. ABN was responsible for data analysis and writing. RTM was responsible for data analysis, writing, and quality assurance. All authors contributed equally planning the research and to the manuscript preparation.
